# Nintedanib inhibits intrahepatic cholangiocarcinoma aggressiveness via suppression of cytokines extracted from activated cancer-associated fibroblasts

**DOI:** 10.1038/s41416-020-0744-7

**Published:** 2020-02-04

**Authors:** Takahiro Yamanaka, Norifumi Harimoto, Takehiko Yokobori, Ryo Muranushi, Kouki Hoshino, Kei Hagiwara, Dolgormaa Gantumur, Tadashi Handa, Norihiro Ishii, Mariko Tsukagoshi, Takamichi Igarashi, Hiroshi Tanaka, Akira Watanabe, Norio Kubo, Kenichiro Araki, Ken Shirabe

**Affiliations:** 1 Department of General Surgical Science, Division of Hepatobiliary and Pancreatic Surgery, Gunma University, Graduate School of Medicine, Gunma, Japan; 2 Gunma University Initiative for Advanced Research (GIAR), Gunma, Japan; 3 Department of Diagnostic Pathology, Gunma University, Graduate School of Medicine, Gunma, Japan; 4 Department of Social Welfare, Gunma University of Health and Welfare, Gunma, Japan; 5 Department of Innovative Cancer Immunotherapy, Gunma University, Graduate School of Medicine, Gunma, Japan

**Keywords:** Cancer microenvironment, Chemotherapy

## Abstract

**Background:**

Intrahepatic cholangiocarcinoma (ICC) is a malignancy that is challenging to treat. Fibroblasts in ICC tissues have been identified as cancer-associated fibroblasts (CAFs) that promote the malignant behaviour of ICC cells. An antifibrotic drug nintedanib has been reported to suppress activated hepatic stellate cells in liver fibrosis.

**Methods:**

We investigated whether nintedanib could suppress the cancer-promoting effect of CAFs derived from ICC tissues in vitro and in vivo.

**Results:**

CAFs promoted the proliferation and invasion of ICC cells. Nintedanib suppressed activated CAFs expressing α-smooth muscle actin (α-SMA) and inhibited the ICC-promoting effects of CAFs. Nintedanib greatly reduced the levels of cancer-promoting cytokines, such as interleukin (IL)-6 (IL-6) and IL-8, secreted by CAFs. An in vivo study demonstrated that nintedanib reduced xenografted ICC growth and activated CAFs expressing α-SMA, and that combination therapy with nintedanib and gemcitabine against CAFs and ICC cells showed the strongest inhibition of tumour growth compared with the control and single-treatment groups.

**Conclusions:**

Nintedanib inhibited the cancer-promoting effect of CAFs via the suppression of CAF activation and secretion of cancer-promoting cytokines. Our findings suggest that therapeutic strategies combining conventional cytotoxic agents with nintedanib targeting CAFs are promising for overcoming refractory ICC with activated CAFs.

## Background

Intrahepatic cholangiocarcinoma (ICC) is the second most common hepatic carcinoma, and its incidence has increased in recent years.^1,2^ Despite recent advances in surgical techniques and chemotherapy, the prognosis of ICC remains poor.^3–6^ Therefore, novel therapeutic strategies are required for improving the prognosis of ICC.

Compared with hepatocellular carcinoma (HCC), ICC presents with extensive fibrosis in the tumour.^7^ Tumour fibrosis is caused by cancer-associated fibroblasts (CAFs), which are reported to play a key role in the ICC microenvironment, and promote malignant behaviour such as proliferation, invasion, metastasis and resistance to therapy via interactive signalling pathways.^8–11^ Indeed, some researchers have reported that CAF-secreted cytokines, such as interleukin (IL)-6 (IL-6), IL-8, C–C motif chemokine ligand 2 (CCL2) and C–X–C motif chemokine ligand 12 (CXCL12), can increase the aggressiveness of co-cultured cancer cells.^12–14^ Moreover, a high expression of α-smooth muscle actin (α-SMA), a marker of activated CAFs, has been correlated with shorter survival times in patients who underwent surgical resection of ICC.^9,10^ These findings suggest that targeting CAFs could be a novel strategy for controlling ICC aggressiveness caused by activated CAFs.

The FDA-approved antifibrotic drug nintedanib is a small-molecule inhibitor of multiple tyrosine kinases, such as fibroblast growth factor receptor (FGFR), vascular endothelial growth factor receptor (VEGFR) and platelet-derived growth factor receptor (PDGFR),^15^ and reportedly, it is therapeutically effective in idiopathic pulmonary fibrosis.^16–18^ It was previously reported that nintedanib evidently suppressed chemical-induced liver fibrosis via the suppression of activated hepatic stellate cells expressing α-SMA, which is a marker of activated-stellate cells.^19^ Moreover, nintedanib was able to suppress the activation of CAFs expressing α-SMA in lung adenocarcinoma.^20^ Therefore, we hypothesised that nintedanib treatment can inhibit the activation of CAFs expressing α-SMA in ICC. However, to the best of our knowledge, studies investigating the effects of nintedanib on CAFs derived from ICC tissues have not yet been conducted.

The aim of the present study was to clarify whether nintedanib could inhibit CAFs derived from ICC tissues. Therefore, we investigated the effects of nintedanib on ICC-derived CAFs that promoted the malignant behaviour of ICC cells, such as proliferation and invasion. In addition, by using an in vivo xenograft model co-implanted with CAFs and ICC cells, we assessed the therapeutic efficacy of combining conventional cytotoxic agents, such as gemcitabine, with nintedanib to target the activated CAFs.

## Methods

### Cell cultures and cell isolation

We established primary CAFs (CAF1 and CAF2) from surgically resected ICC tissues in Gunma University, using the method described by Lau et al.^21^ Briefly, ICC tissues were minced into small pieces and incubated in Dulbecco’s modified Eagle medium (DMEM, Wako, Osaka, Japan) supplemented with 20% foetal bovine serum (FBS), 1% penicillin–streptomycin (Thermo Fisher Scientific, Kanagawa, Japan) and 1 ng/mL basic fibroblast growth factor (bFGF, Wako), and were maintained at 37 °C in a humidified incubator with 5% CO_2_ to allow attachment to the culture plate. We then removed the unattached cells, and replenished the medium repeatedly twice a week. We confirmed that the CAFs exhibited a myofibroblast-like morphology; moreover, the positive expression of α-SMA was confirmed using Western blotting. For our experiments, we used the CAFs between passage numbers 4 and 8. The establishment of CAFs from surgically resected ICC tissues was approved by the Institutional Review Board of Gunma University (Approval number: 2016-118). The human ICC cell lines, HuCCT1 and RBE, were used in the present study. The HuCCT1 cell line was provided by RIKEN BRC (Bio-Resource Center) in Japan, and authenticated by short tandem repeat DNA profiling by BEX Co., Ltd. (Tokyo, Japan). The RBE cell line was also provided by RIKEN BRC. Cells were cultured in DMEM supplemented with 10% FBS and 1% penicillin–streptomycin (Thermo Fisher Scientific, Kanagawa, Japan), and maintained at 37 °C in a humidified incubator with 5% CO_2_ atmosphere.

### Nintedanib (BIBF1120)

Nintedanib used in the present study was purchased from Selleck (TX, USA) and Med Chem Express (NJ, USA). Briefly, nintedanib was dissolved in dimethyl sulfoxide (DMSO, Wako, Osaka, Japan) at a final concentration of 1–10 nM/mL for the in vitro experiments. Nintedanib was dissolved in Tween-80 (Sigma-Aldrich) solution at a final concentration of 30 mg/kg for the in vivo experiments.

### Protein extraction and Western blotting

CAFs were treated with nintedanib at concentrations varying between 0 and 3 µM, and 5 ng/mL recombinant transforming growth factor β (TGFβ, R&D systems, Minneapolis, USA) for 72 h followed by protein extraction. ICC cell lines were treated with nintedanib, CAF-conditioned medium (CM) and nintedanib-treated CAF–CM for 12 h, followed by protein extraction. Cells of the ICC cell line were treated with 10 ng/ml recombinant IL-6 Protein (R&D systems) and 10 ng/ml recombinant IL-8 Protein (R&D systems) for 2 h, followed by protein extraction. Protein extraction was performed using RIPA buffer (Wako) containing phosphatase inhibitor (p2850, Sigma-Aldrich) according to the manufacturer’s instructions. Proteins were separated using SDS-PAGE with 10% Bis–Tris gels, and transferred to nitrocellulose membranes (#12369, Cell Signaling). Membranes were blocked with 5% skimmed milk or 5% BSA, and incubated overnight at 4 °C with the primary antibodies. The primary antibodies used in the present study were anti-α-SMA mouse monoclonal antibody (A2547, 1:1000, Sigma-Aldrich, St. Louis, MO, USA), Phospho-STAT3 rabbit antibody (#9145, 1:1000, Cell Signaling Technology, MA, USA), STAT3 mouse antibody (#9139S, 1:1000, Cell Signaling Technology) and anti-β-actin mouse monoclonal antibody (A5316, 1:1000, Sigma-Aldrich) as a loading control. Next, membranes were treated with horseradish peroxidase (HRP)-conjugated secondary antibodies. Protein bands on the membrane were detected using ECL™ Prime Western Blotting Detection Reagent and an ImageQuant™ LAS 4000 imager (GE Healthcare, Buckinghamshire, UK).

### Conditioned medium

CAFs were cultured in DMEM supplemented with 20% FBS and 1 ng/mL bFGF, until they reached a confluence of 90%. Next, the medium was changed to serum-free DMEM, and the cells were cultured for an additional 120 h. At the end of the incubation, CAF–CM was collected and centrifuged for 10 min at 1500 *g*. In order to evaluate the effect of nintedanib, CAFs were cultured in serum-free DMEM containing 1 µM nintedanib for 120 h, and collected as nintedanib-treated CAF–CM.

### Ultracentrifugation

Ultracentrifugation was performed as previously described.^13^ Briefly, CAF–CM was ultracentrifuged at 110,000 × *g* for 70 min at 4 °C. The supernatant was collected after the first centrifugation. The pellets were washed with phosphate-buffered saline, subjected to ultracentrifugation and resuspended in serum-free DMEM.

### Cell proliferation assay

The cell proliferation assay was performed using a Cell Counting Kit-8 (CCK-8, Dojindo Laboratories, Kumamoto, Japan). The CAFs and ICC cell lines were seeded in 96-well plates and cultured. Culture medium was changed to serum-free medium containing nintedanib at concentrations varying between 0 and 10 µM. To assess cell proliferation after stimulation with CM, the medium of ICC cells was also replaced with CAF–CM or nintedanib-treated CAF–CM after overnight incubation. Cell proliferation was evaluated after 48 h. The absorbance of each well was measured using a spectrophotometer (Bio-Rad, Hercules, CA, USA) at 450 nm with the reference wavelength set at 650 nm.

### Invasion assay

The cell invasion assay was performed using 24-well Corning^®^ BioCoat™ Matrigel Invasion Chambers (Corning, NY, USA). ICC cell lines were seeded in the upper chamber in FBS-free medium, and the lower chamber was filled with medium supplemented with 3% FBS and containing 1 µM nintedanib, CAF–CM or nintedanib-treated CAF–CM. After incubation for 48 h, the cells were fixed and stained with Diff-Quik (Sysmex Corporation, Kobe, Japan). After staining, cells that migrated through the pores to the lower surface of the membrane were counted under the microscope. In total, five randomly selected fields were evaluated.

### Cytokine array and enzyme-linked immunosorbent assay (ELISA)

Cytokine profiles of cells treated with CAF–CM and nintedanib-treated CAF–CM were compared using the Proteome Profiler Human XL Cytokine Array Kit (ARY022B, R&D Systems, MN, USA), according to the manufacturer’s instructions. Detection and quantification of the array spots were performed using the ImageQuant™ LAS 4000 imager (GE Healthcare). Concentrations of cytokines in CAF–CM and nintedanib-treated CAF–CM-treated cells were measured using the enzyme-linked immunosorbent assay (ELISA). The IL-6 (ab46027) and IL-8 (ab46032) ELISA kits were purchased from Abcam (Cambridge, MA, USA) and used according to the manufacturer’s instructions.

### In vivo experiments

First, to investigate the effect of CAFs in an in vivo xenograft model, we compared the proliferation rates of HuCCT1 cells alone and HuCCT1 plus CAF cells. HuCCT1 cell suspensions (5 × 10^6^ cells) and HuCCT1 (5 × 10^6^ cells) plus CAF (1 × 10^6^ cells) cell suspensions were bilateral and subcutaneously injected into the flanks of isoflurane-anesthetised 7-week-old female NOD-SCID mice (CLEA Japan, Inc., Tokyo, Japan). Each group contained five xenografts. The experiment was conducted as per the schedule given in Supplementary Fig. 1A. Moreover, to analyse the effects of nintedanib in vivo, we used a mouse xenograft model of HuCCT1 plus CAF cells. Two weeks after implantation, we randomly divided mice into the control, nintedanib, gemcitabine and nintedanib and gemcitabine groups. We administered 30 mg/kg nintedanib orally five times a week, and 50 mg/kg gemcitabine intraperitoneally twice a week to mice under isoflurane anaesthesia. Each group contained six xenografts. The experiment was conducted as per the schedule given in Supplementary Fig. 1B. Tumour diameters and body weights were measured twice a week, and tumour volume was calculated using the following formula: S × S × L/2, where S is the short diameter of the tumour, and L is the long diameter of the tumour. To collect xenografted tumours, mice were anaesthetised with isoflurane and euthanised via cervical dislocation. The tumours were microscopically evaluated using haematoxylin and eosin staining and immunohistochemistry. To evaluate the adverse events of treatment, we obtained blood samples from mice; serum biochemical tests were conducted by the Oriental Yeast Co. (Tokyo, Japan). Mice had free access to water and food, and were housed in specific pathogen-free cages and bedding in a 12-h light/dark regimen with controlled room temperature. All mouse experiments were performed in compliance with the guidelines of the Institute for Laboratory Animal Research at Gunma University, Maebashi, Japan (Approval number: 18-024).

### Immunohistochemistry

Immunohistochemistry analysis of tumour samples was performed using the following primary antibodies: mouse monoclonal anti-α-SMA antibody (A5247, 1:1000, Sigma-Aldrich), anti-Ki-67 antibody (M7240, 1:200, Dako, Agilent Technologies, CA, USA) and phospho-STAT3 rabbit antibody (#9145, 1:200, Cell Signaling Technology). The amount of α-SMA- and Ki-67-positive cells were measured using the ImageJ 1.51 image analysis software (National Institute of Health, MD, USA). The number of phospho-STAT3-positive cells was counted under the microscope.

### Statistical analysis

Data for continuous variables are expressed as means ± standard deviation (SD). Differences between two groups were estimated using *t* tests. Differences among four groups were evaluated using ANOVA with Tukey’s multiple comparison tests. All *p* < 0.01 or 0.05 were considered statistically significant. All statistical analyses were performed using the statistics software Easy R (EZR).

## Results

### ICC-derived CAFs promote proliferation and invasion of ICC cells in vitro

**Fig. 1:**
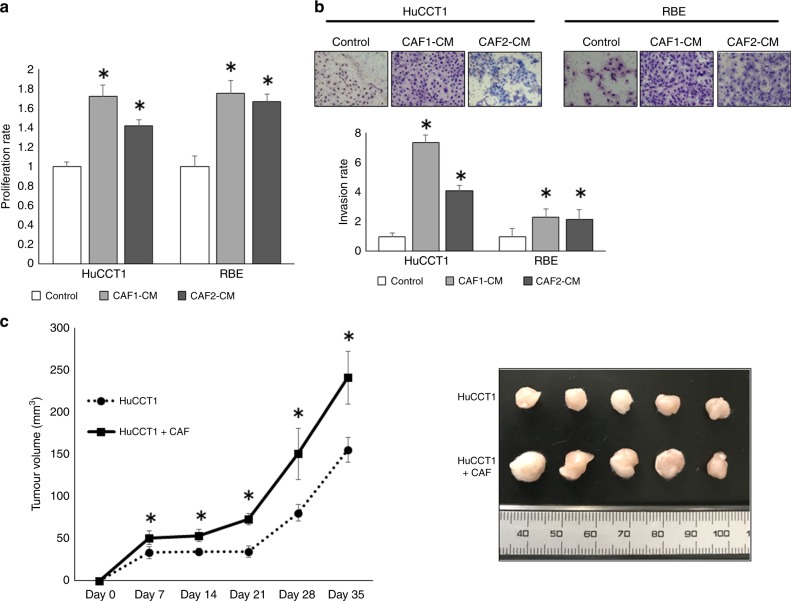
Effects of CAFs on ICC cells in vitro and in vivo. **a** CM from CAF1 and CAF2 enhances the proliferation of both HuCCT1 and RBE (*n* = 5). *P < 0.01. **b** CM from CAF1 and CAF2 enhances the invasive ability of both HuCCT1 and RBE (n = 5). *P < 0.01. **c** The tumour growth curve of HuCCT1 and HuCCT1 plus CAF cells. The tumour growth of HuCCT1 + CAFs was much greater compared with that of HuCCT1 alone (*n* = 5). *P < 0.05.

In order to confirm the effect of CAFs on cancer progression, we evaluated the cancer proliferation and invasion of ICC cell lines treated with CAF–CM. CAF–CM promoted the proliferation and invasion of ICC cell lines (Fig. 1a, b). Moreover, the tumour volume of HuCCT1 cells co-implanted with CAFs grew to a size larger than those of HuCCT1 cells alone in vivo (Fig. 1c).

### Antifibrotic drug nintedanib suppresses the activation of CAFs

**Fig. 2:**
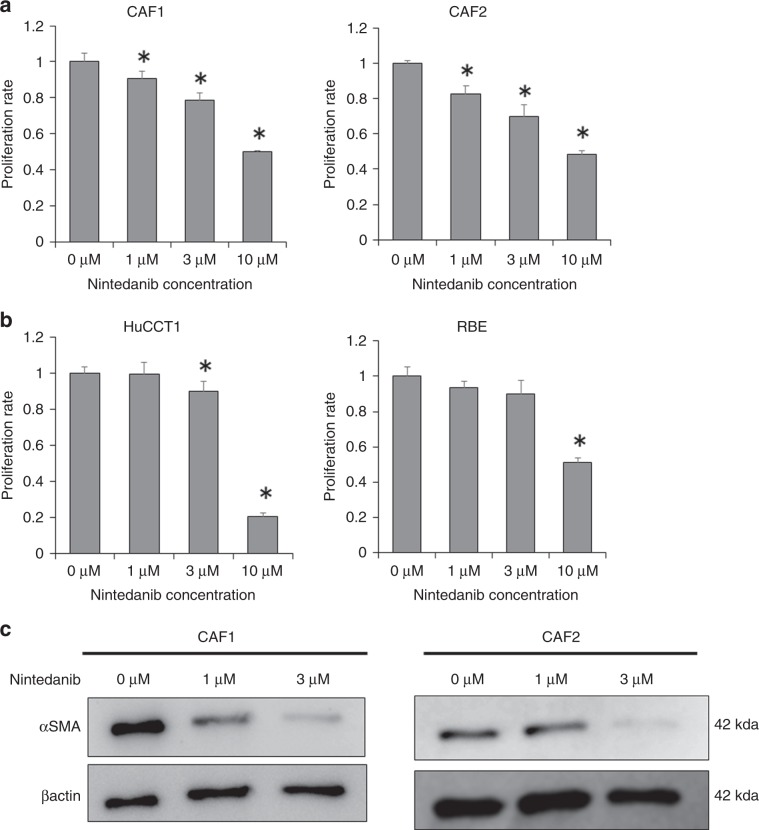
Effects of nintedanib on CAFs and ICC cells. **a** Nintedanib inhibits the proliferation of CAFs concentration-dependently (*n* = 5). *P < 0.01. **b** Nintedanib inhibits the proliferation of HuCCT1 and RBE only at high concentrations (*n* = 5). *P < 0.01. **c** α-SMA expression in CAFs treated with nintedanib is evaluated by western blotting. CAFs were pre-activated with 5 ng ml^−1^ TGF-b1. β-actin is used as the loading control. *P < 0.01.

In order to examine the effect of nintedanib on CAFs and ICC cell lines, we evaluated the effect of nintedanib on the proliferation of CAFs and ICC cell lines. Nintedanib significantly suppressed CAF proliferation in a concentration-dependent manner compared with the control (Fig. 2a). On the other hand, the suppression effect of nintedanib against ICC cell lines was observed only at the high concentrations of 3 or 10 µM (Fig. 2b). Moreover, nintedanib suppressed the expression of α-SMA, an activated CAF marker, in CAF1 and CAF2 cells in a concentration-dependent manner (Fig. 2c).

### Nintedanib could eliminate the CAF-induced proliferative and invasive abilities of ICC cells

**Fig. 3:**
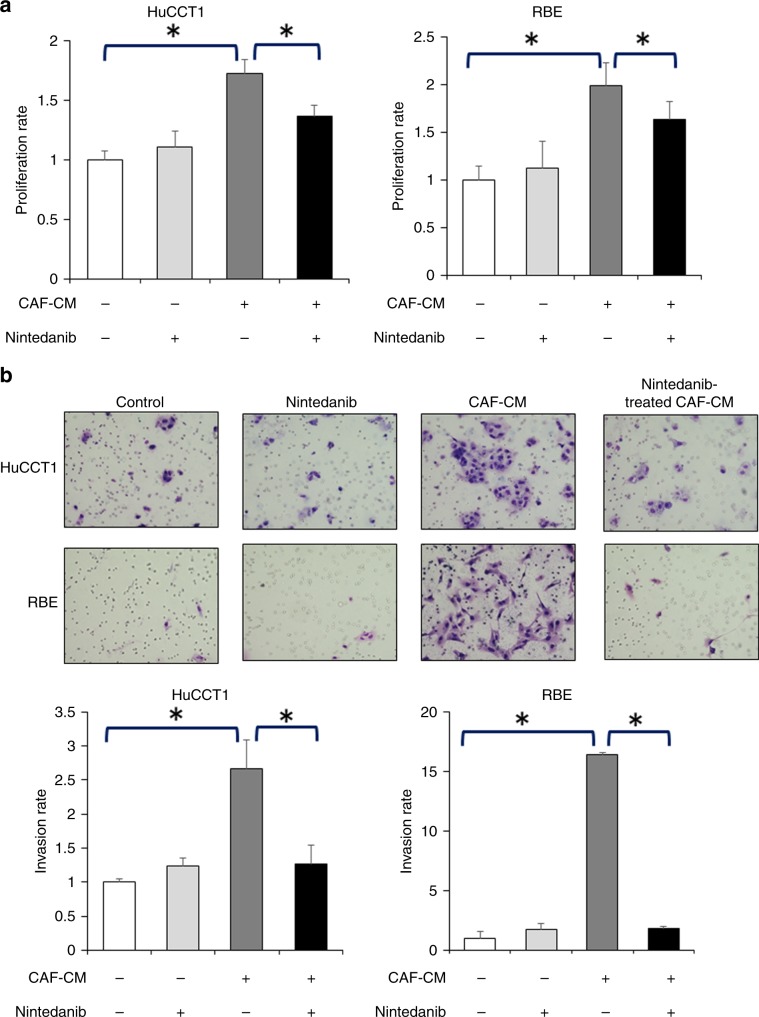
Nintedanib inhibits the ICC-promoting effects of CAFs. **a** The proliferation-enhancing effects of CAF–CM on HuCCT1 and RBE are eliminated by nintedanib treatment (*n* = 5). *P < 0.01. **b** The invasiveness-enhancing effects of CAF–CM on HuCCT1 and RBE are decreased by nintedanib treatment (*n* = 5). *P < 0.01.

In order to determine the effects of nintedanib on the malignant behaviour of ICC cell lines induced by CAFs, we investigated the proliferative and invasive capabilities of the ICC cell lines using nintedanib-treated CAF–CMs. As mentioned above, there was no significant direct effect of a low (1 µM) nintedanib concentration on the proliferation and invasion of ICC cell lines (Fig. 3a, b), and the CAF–CM increased the proliferation and invasion of ICC cells. Interestingly, the proliferation-enhancing effects of CAF–CM were eliminated by nintedanib treatment (Fig. 3a), and it was observed that nintedanib could decrease CAF–CM- enhanced ICC cell line invasion (Fig. 3b).

### Nintedanib suppressed cytokine secretion by CAFs

**Fig. 4:**
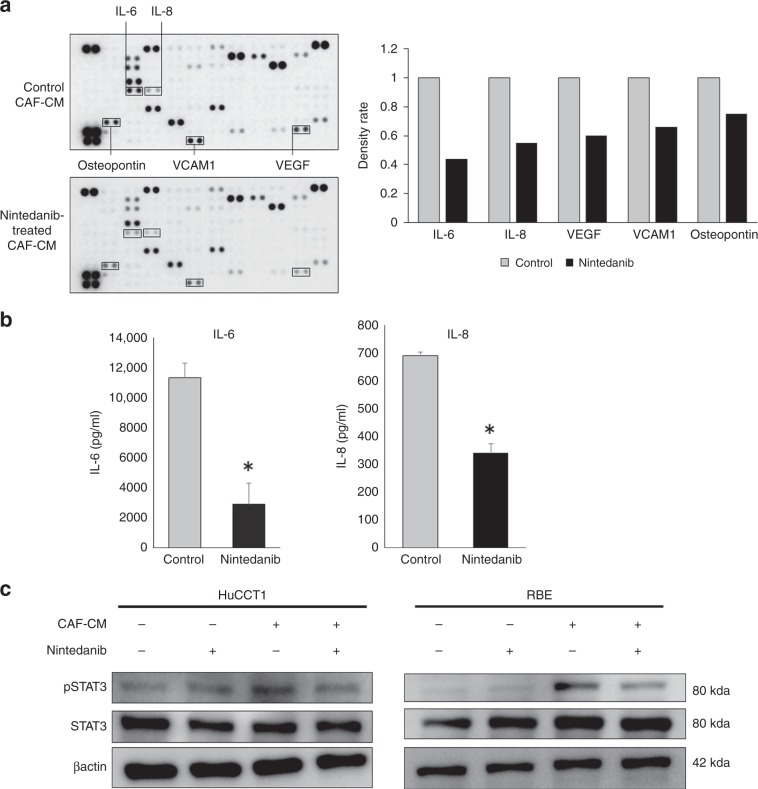
Nintedanib suppresses the cytokine secretion of CAFs. **a** Cytokine array comparing CAF–CM and nintedanib-treated CAF–CM. IL-6, IL-8, VEGF, VCAM1 and osteopontin were reduced by nintedanib treatment. The right panel shows the density ratio of array dots between CAF–CM and nintedanib-treated CAF–CM. Cytokine array was performed using CAF1. **b** ELISA of IL-6 and IL-8 in CAF–CM and nintedanib-treated CAF–CM. IL-6 and IL-8 were reduced by nintedanib treatment (*n* = 5). *P < 0.01. **c** The effects of CAF–CM and nintedanib-treated CAF–CM on the phosphorylation of STAT3 in HuCCT1 and RBE cell lines were evaluated by Western blotting. CAF–CM increased the phosphorylation of STAT3 in ICC cell lines, but the increasing effects were eliminated by nintedanib treatment.

To determine the factors involved in CAF suppression by nintedanib, we used ultracentrifugation to allow the evaluation of soluble factors such as cytokines, and the pellet after ultracentrifugation, which might include the extracellular vesicles. The supernatants of CAF–CM after ultracentrifugation promoted the proliferation of cancer cells. This effect was eliminated by treatment with nintedanib, as with the original solutions. Conversely, the resuspended pellets did not exhibit any significant effect on the cancer cells (Supplementary Fig. 2). Subsequently, we performed cytokine array analysis to evaluate the differences between control CAF–CM and CAF–CM treated with nintedanib for 105 types of soluble factors, including cytokines and proteins (Supplementary Table 1). Although various cytokines and proteins were suppressed by nintedanib treatment, interleukin-6 (IL-6), interleukin-8 (IL-8), vascular endothelial growth factor (VEGF), vascular cell adhesion molecule 1 (VCAM1) and osteopontin were remarkably decreased in the nintedanib-treated CAF–CM compared with the control (Fig. 4a). Among these, we focused on IL-6 and IL-8 as the cytokines that were significantly suppressed by nintedanib, and we validated the significant suppression of IL-6 and IL-8 in nintedanib-treated CAF–CM using ELISA (Fig. 4b). Moreover, we confirmed the effects of CAF–CM and nintedanib-treated CAF–CM on the phosphorylation of STAT3, which is known as a representative downstream target of several external cytokines including IL-6 and IL-8. CAF–CM remarkably increased the phosphorylation of STAT3 in ICC cell lines, but the increasing effects on CAF phosphorylation were eliminated by nintedanib treatment (Fig. 4c). We confirmed that treatment with recombinant IL-6 and IL-8 protein promoted the phosphorylation of STAT3 (Supplementary Fig. 3A). Moreover, we confirmed that nintedanib-treated CAF–CM with recombinant IL-6 and IL-8 protein restored the cancer-promoting effects of CAFs (Supplementary Fig. 3B).

### Nintedanib inhibits tumour proliferation, and combination therapy with gemcitabine inhibits tumour proliferation even more remarkably

**Fig. 5:**
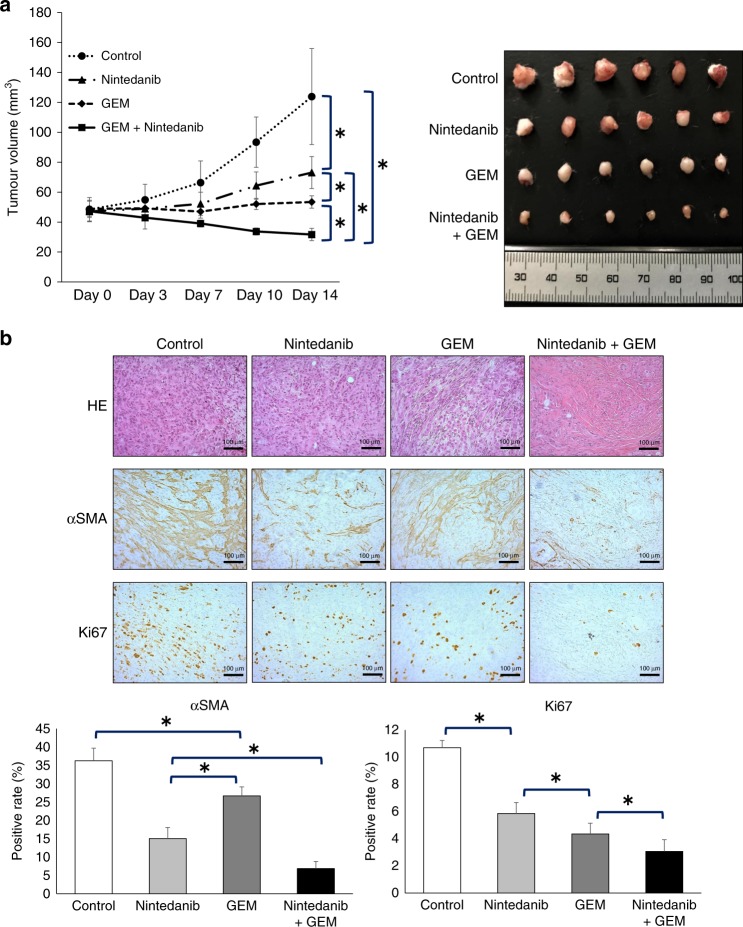
Nintedanib reduces tumour volume consisting of HuCCT1 plus CAFs, and combination therapy with gemcitabine inhibits tumour proliferation markedly. **a** Tumour growth curve shows that combination therapy with nintedanib plus gemcitabine suppresses the growth of tumours consisting of HuCCT1 plus CAFs to the greatest extent. Photograph of tumours from the four treatment groups (control, nintedanib, gemcitabine and nintedanib + gemcitabine). GEM, gemcitabine (*n* = 6). *P < 0.05. **b** Representative photomicrographs of histological examinations comparing the four treatment groups (control, nintedanib, gemcitabine and nintedanib + gemcitabine) (HE, α-SMA and Ki-67, original magnification ×200, scale bar 100 µm). Nintedanib treatment significantly reduced α-SMA-positive staining in stroma. Gemcitabine treatment significantly reduced the proportion of Ki-67-positive cells. Nintedanib plus gemcitabine treatment showed a reduction of both α-SMA-positive staining in stroma and the proportion of Ki-67-positive ICC cells. GEM, gemcitabine (*n* = 5). *P < 0.05.

We investigated the combined effect in vivo of nintedanib and gemcitabine, which is a standard therapeutic drug against ICC in clinical practice. Although treatment with nintedanib alone and gemcitabine alone inhibited tumour proliferation, therapy with nintedanib and gemcitabine combined inhibited tumour growth to an even greater degree than with each agent. In addition, tumour shrinkage, as compared with the tumour volume on day 0, was only observed in the nintedanib and gemcitabine combination therapy group (Fig. 5a). The tumours treated with nintedanib showed a reduction of α-SMA-positive staining area compared with controls or those treated with gemcitabine alone (Fig. 5b). The proportion of Ki-67-positive ICC cells was significantly lower in tumours treated with gemcitabine compared with controls or those treated with nintedanib (Fig. 5b). Tumours treated with nintedanib and gemcitabine combined showed a reduction of both the α-SMA-positive staining area and the proportion of Ki-67-positive ICC cells (Fig. 5b). Nintedanib treatment resulted in a significant reduction in the number of pSTAT3-positive cells, with the greatest reduction observed with combined nintedanib and gemcitabine treatment (Supplementary Fig. 4). Although we evaluated adverse events associated with the treatments in vivo, there were no significant differences among the groups regarding body weight and organ toxicity (Supplementary Fig. 5A, B).

## Discussion

Herein, we have demonstrated that CAFs promoted ICC malignancy in vitro and in vivo, and nintedanib treatment suppressed the ability of CAFs to enhance the aggressiveness of ICC cells. This study is the first report to show the CAF suppression effect of nintedanib in vivo. We also showed that various cytokines produced by CAFs, such as IL-6, IL-8, VEGF, VCAM1 and osteopontin, were strongly suppressed by the treatment with the antifibrotic drug nintedanib. Moreover, in vivo experiments showed that nintedanib suppressed the α-SMA-positive staining area in tumours, and that combination therapy with gemcitabine inhibited tumour growth to an even greater extent compared with single-agent treatment. It was suggested that targeting strategy against both CAFs by nintedanib and ICC cells by gemcitabine is a useful therapeutic tool to treat refractory ICC with activated CAFs.

Recently, tumour–stromal interactions in cancer tissues have been recognised as playing important roles in tumour progression, and have been highlighted in several reviews.^9–11^ To target the tumour–stromal interactions, anticancer therapy against not only cancer cells but also stromal CAFs has actively been investigated. Recent studies have shown that navitoclax,^22^ resveratrol^12^ and everolimus^23^ suppressed CAFs in cholangiocarcinomas. Previously, we reported that the antifibrotic drug conophylline, a natural vinca alkaloid compound, could inhibit pancreatic cancer progression through the suppression of CAFs originated from stellate cells in pancreatic cancer.^13^ Consistent with our previous findings, some researchers reported the fundamental effect of conophylline as an inhibitor of activated-stellate cells in the liver and pancreas.^24,25^ In this study, we focused on the effect of the FDA-approved, antifibrotic drug nintedanib against CAFs derived from ICC tissues. Nintedanib is an inhibitor of multiple tyrosine kinases, such as FGFR, VEGFR and PDGFR, and it has shown therapeutic effects against stromal fibrosis in patients with idiopathic pulmonary fibrosis.^16–18^ It was previously reported that nintedanib clearly suppressed chemical-induced liver fibrosis through the suppression of activated hepatic stellate cells with α-SMA, which is a marker of activated-stellate cells.^19^ In this study, antifibrotic drug nintedanib suppressed activated CAFs with α-SMA, which could enhance the aggressiveness of ICC cells. From these observations, antifibrotic drugs such as nintedanib may be candidates for the inhibition of tumour–stromal interaction, including cancer cells and CAFs via suppression of activated CAFs with α-SMA.

Nintedanib has been shown to inhibit the proliferation and activation of lung fibroblasts and hepatic stellate cells.^18,19^ Moreover, nintedanib has been shown to inhibit the tumour-promoting effect of CAFs in lung adenocarcinoma using CAF–CMs in vitro.^20^ The activation of PDGF/PDGFR and FGF/FGFR signalling has been reported to significantly induce the proliferation and activation of fibroblasts including CAFs;^26–28^ however, as mentioned above, nintedanib has been known to inhibit PDGFR and FGFR.^15^ Moreover, it was reported that cholangiocarcinoma cells stimulated migration of CAFs via PDGF secretion.^29^ In the present study, we showed that nintedanib suppressed the proliferation and activation of CAFs of ICC in vitro and in vivo. Therefore, it was suggested that nintedanib inhibits the activation of CAFs derived from ICC via suppression of CAF activators such as PDGF/PDGFR and FGF/FGFR signalling pathways.

In a clinical trial aimed to clarify the antitumour effect of nintedanib monotherapy for patients with colorectal cancer, the monotherapy could not improve the overall survival of these patients.^30^ Conversely, nintedanib, in combination with docetaxel, has been used for the second-line treatment in patients with advanced non-small-cell lung cancer.^31^ In the present study, a low concentration (1 µM) of nintedanib could not suppress the proliferation of ICC cell lines (Fig. 2). Nintedanib was suggested to be more effective on CAFs than cancer cells because the low concentration (1 µM) of nintedanib could specifically suppress the CAF activation and secretion of cytokines in vitro. In an in vivo study, administration of nintedanib alone could strongly suppress the stromal α-SMA-positive staining of xenograft tumours, but could not suppress the proportion of Ki-67-positive ICC cells strongly. The results of our study also suggested that nintedanib specifically suppresses the proliferation and activation of CAFs in vivo. Conversely, administration of gemcitabine alone could suppress the proportion of Ki-67-positive ICC cells in a xenograft tumour, but could not suppress the stromal α-SMA-positive staining. Gemcitabine, a representative cytotoxic agent that acts on proliferating cancer cells, was suggested to be effective only on ICC cells with high proliferation potency. Interestingly, the combination treatment with nintedanib and gemcitabine showed tumour shrinkage, as well as reduction of stromal α-SMA-positive staining and Ki-67-positive ICC cells. These data suggested that nintedanib and gemcitabine suppressed both activated CAFs with α-SMA and proliferating ICC cells, respectively. Therefore, combination therapy with nintedanib and cytotoxic agents, such as gemcitabine and docetaxel, may be very effective against cancers with activated CAFs with α-SMA.

In the present study, we showed that nintedanib suppressed the activation of CAFs, and significantly inhibited the secretion of various cytokines, especially IL-6, IL-8, VEGF, VCAM1 and osteopontin. These factors have been reported to play important roles in tumour growth. For instance, IL-6 was reported to regulate JAK–STAT3 signalling that promotes the proliferation of tumour cells, invasion and immunosuppression.^32–35^ IL-8 was reported to promote the proliferation of tumour cells, invasion and metastasis in lung and pancreatic cancer.^14,36,37^ VCAM1 is involved in the micro-environmental interaction between tumour and stromal cells, and promotes the metastasis of breast cancer cells.^38^ VEGF was reported to promote cancer proliferation and angiogenesis.^39,40^ Osteopontin was reported to be a prognostic marker of ICC, and promoted the metastasis of ICC.^41,42^ From the above-mentioned findings, nintedanib might suppress the ICC-promoting effects of CAFs through suppression of these cytokines.

In this study, we showed that nintedanib could suppress cancer-related cytokines, particularly, IL-6, IL-8 and VEGF. IL-6 was reported to induce the chemoresistance in cancer via activation of STAT3.^43^ IL-8 and VEGF were reported to activate STAT3 in cancer cells.^44,45^ The activation of STAT3 seems to be important for chemoresistance in cholangiocarcinoma cells.^46^ Moreover, activated STAT3 was reported to induce resistance to gemcitabine in pancreatic cancer.^47,48^ In this study, we showed that nintedanib not only suppressed the secretion of the cancer-promoting cytokines induced by CAFs, but also the phosphorylation of STAT3 in ICC cells, which is induced by CAFs. Therefore, our results suggest that the combination of nintedanib and gemcitabine can promote the regulation of CAF-secreting cytokines that can cause gemcitabine resistance via activation of STAT3 in ICC cells.

Our study had some limitations. First, we did not perform sorting to collect a pure CAF population using flow cytometric analysis. Therefore, there is a potential limitation of contamination with mesenchymal cells. Second, previous studies have reported that nintedanib can suppress not only CAFs, but also non-cancer-derived fibroblastic cells. We confirmed that nintedanib could suppress proliferation and α-SMA expression of non-cancer-derived fibroblast LX-2 (hepatic stellate cell line) (Supplementary Fig. 6). Therefore, we cannot exclude the possibility of off-target effects against non-cancer-derived fibroblasts in the whole body in the in vivo experiment.

In conclusion, we demonstrated that nintedanib inhibited the cancer-promoting effect of CAFs via suppression of CAF activation and secretion of multiple cancer-promoting cytokines. Nintedanib may be a useful tool to simultaneously regulate the activated CAFs and cancer-promoting cytokines. Moreover, we showed that the combination of gemcitabine and nintedanib, one targeting cancer cells and the other targeting CAFs, respectively significantly reduced the tumour volume in xenografted tumour models. Therefore, combination therapy with nintedanib and cytotoxic agents may be promising to overcome refractory ICC with activated CAFs.

## Supplementary information

Supplementary files

## Data Availability

All data generated or analysed during this study are included in this article.
