# Supplementary material for: Nintedanib inhibits intrahepatic cholangiocarcinoma aggressiveness via suppression of cytokines extracted from activated cancer-associated fibroblasts
Source: Br J Cancer. 2020 Feb 4;122(7):986–94. doi: 10.1038/s41416-020-0744-7 (PMC7109053; doi:10.1038/s41416-020-0744-7)
Supplement: Supplementary file 1 — Supplementary files [file 41416_2020_744_MOESM1_ESM.pdf]

Supplementary Figure 1

A

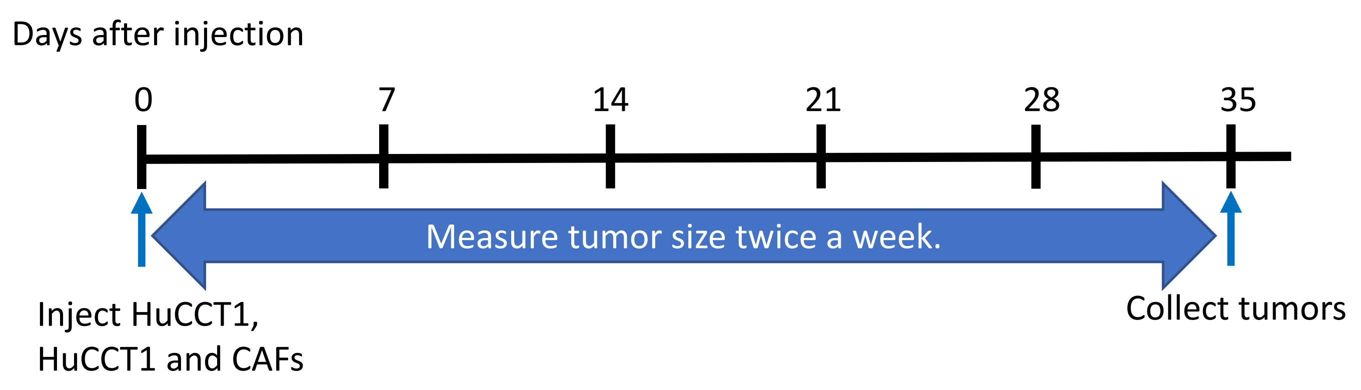

B

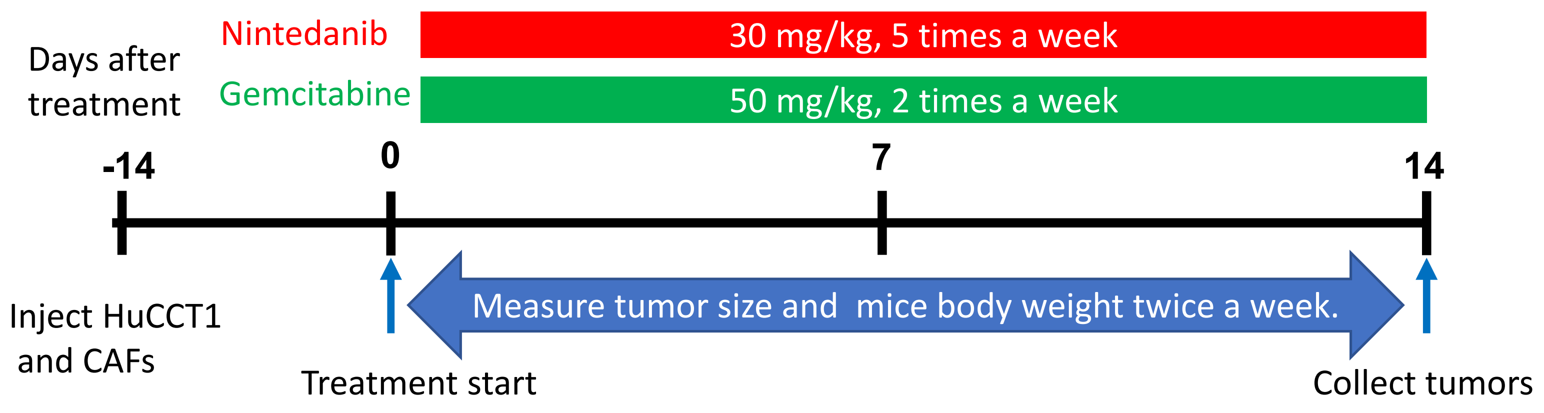

Supplementary Figure 2

A

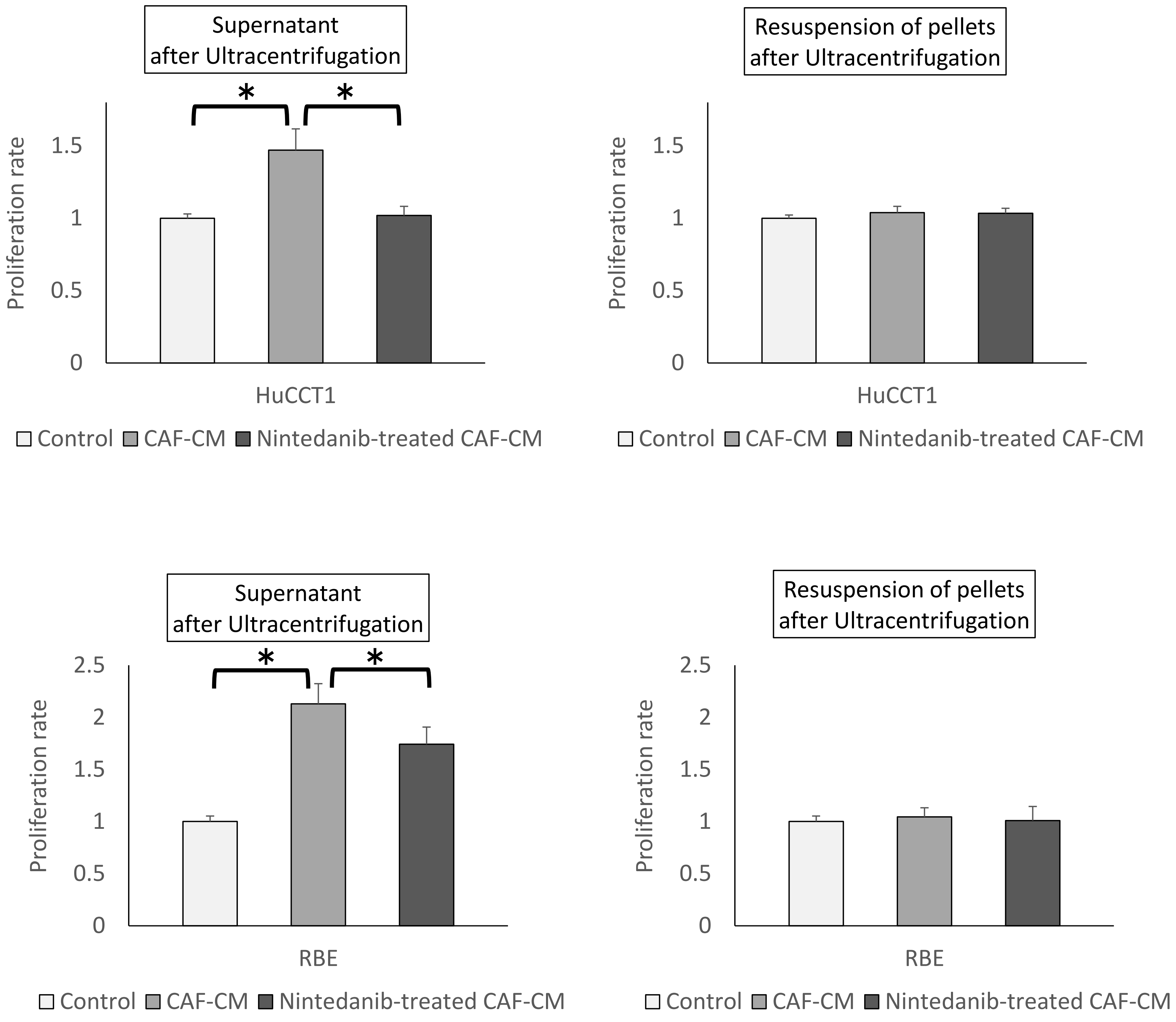

Supplementary Figure 3

A

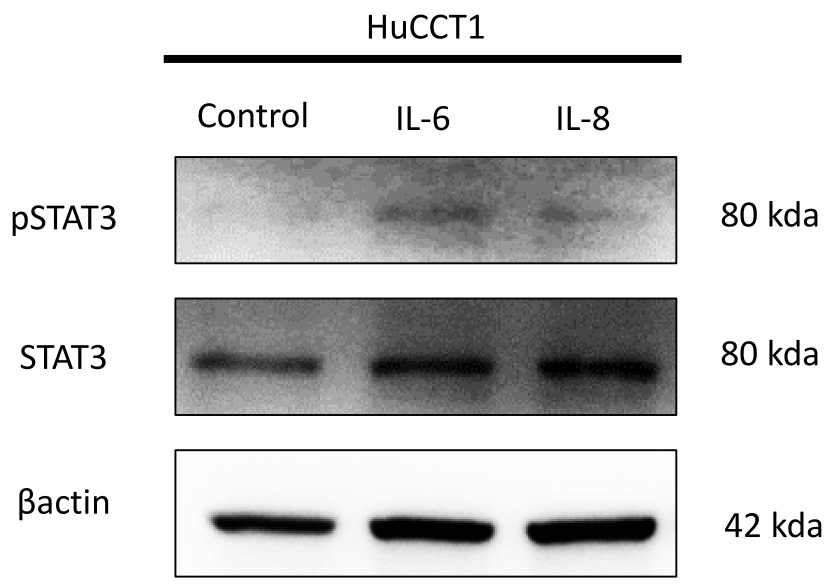

B

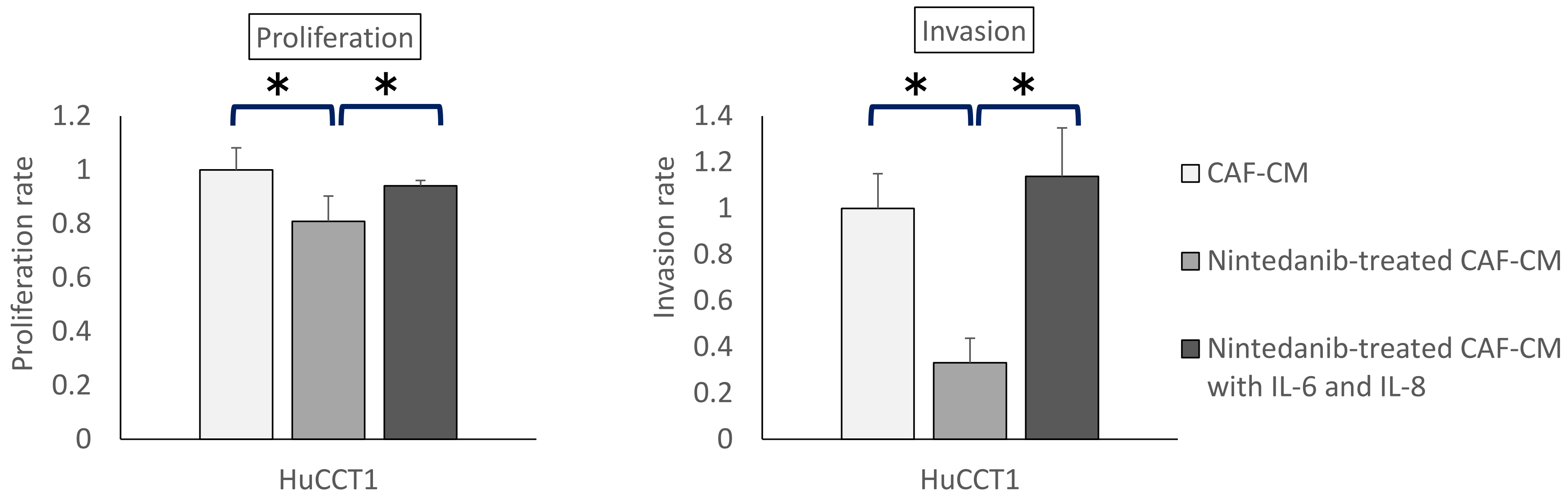

Supplementary Figure 4

A

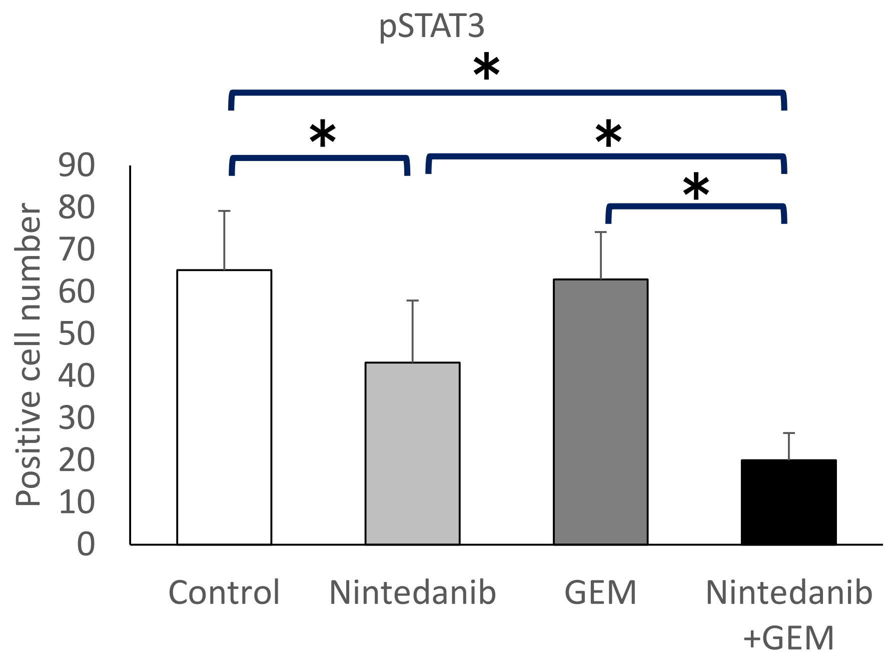

Supplementary Figure 5

A

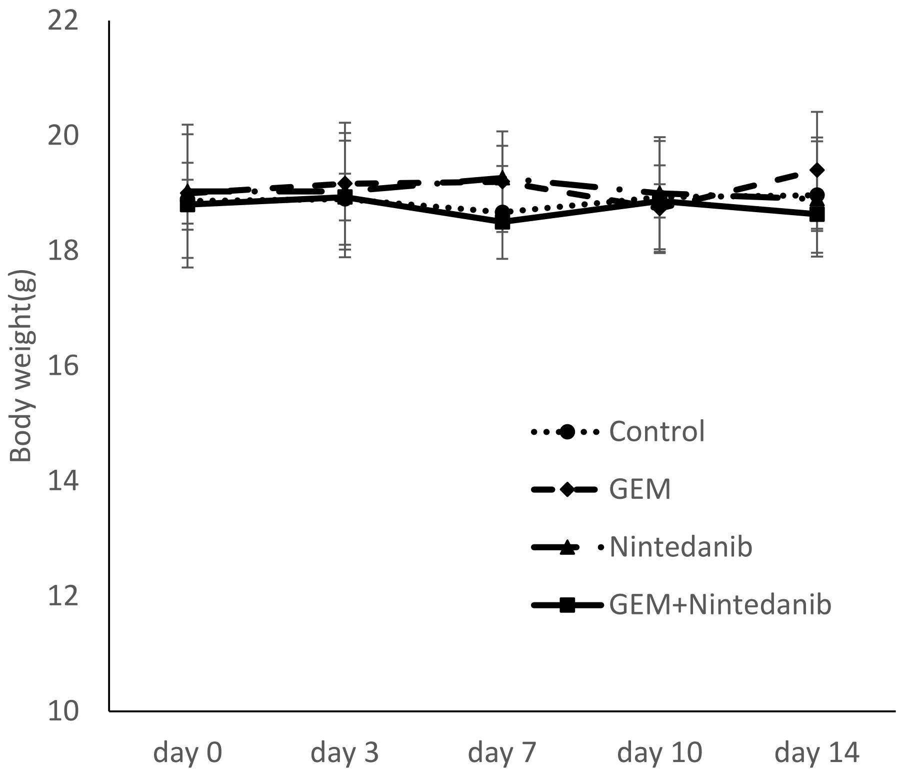

B

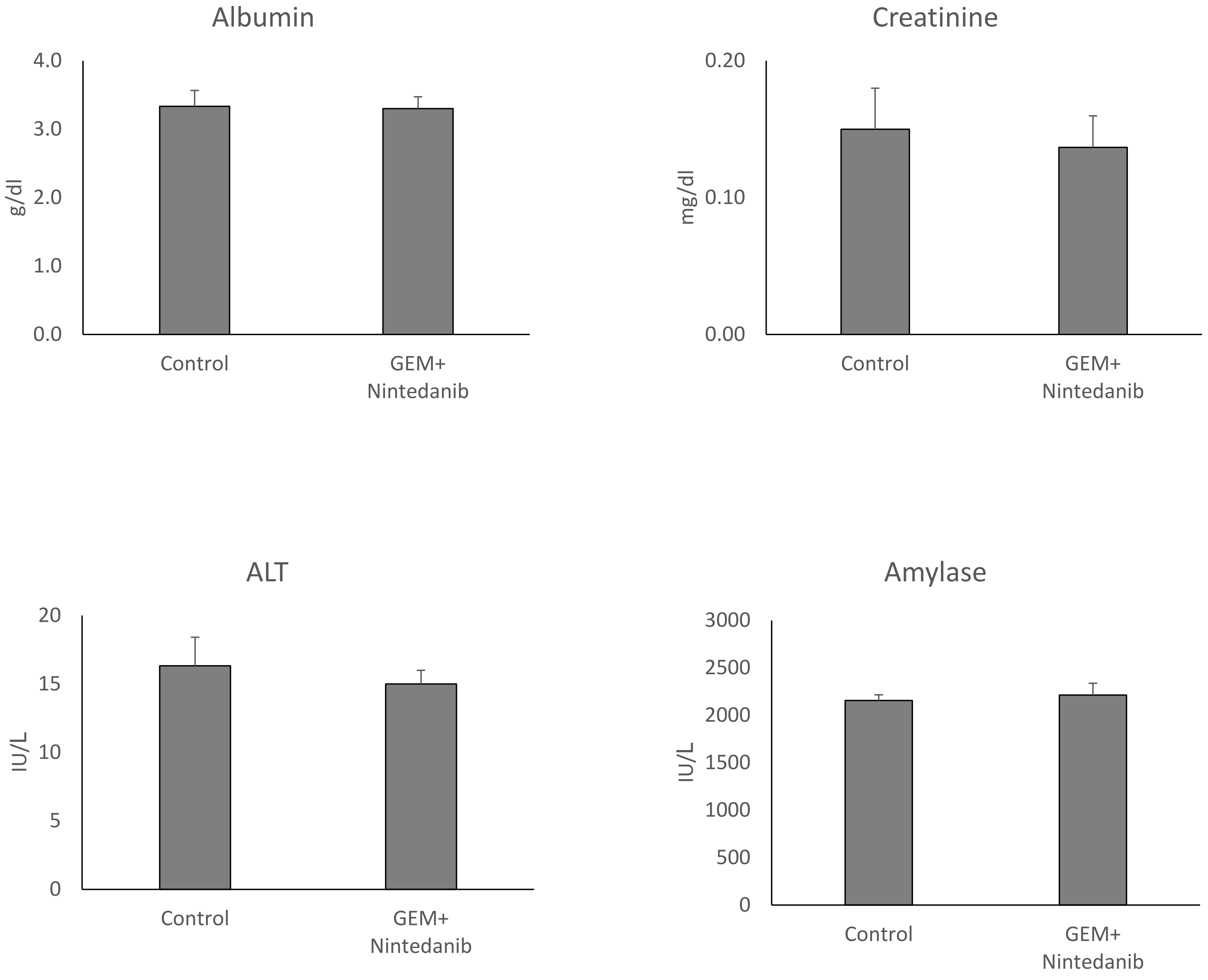

Supplementary Figure 6

A

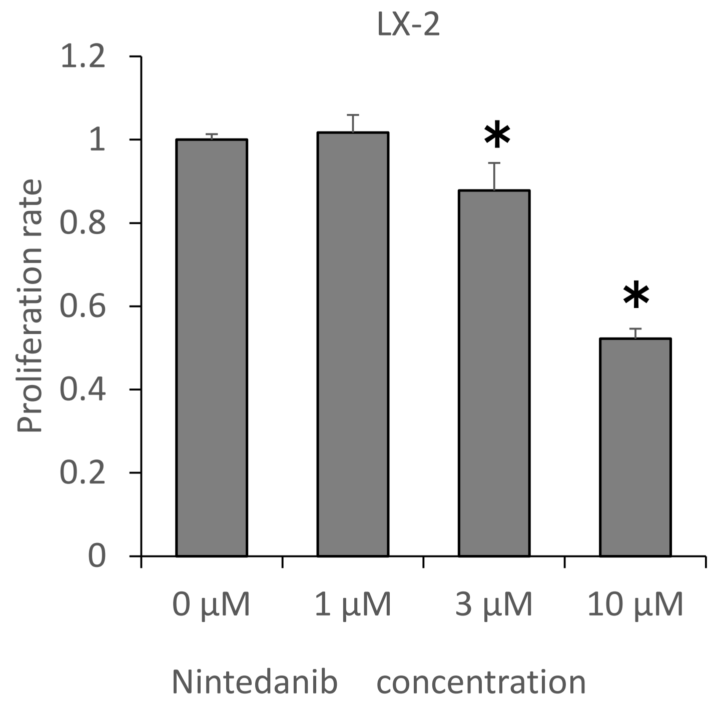

B

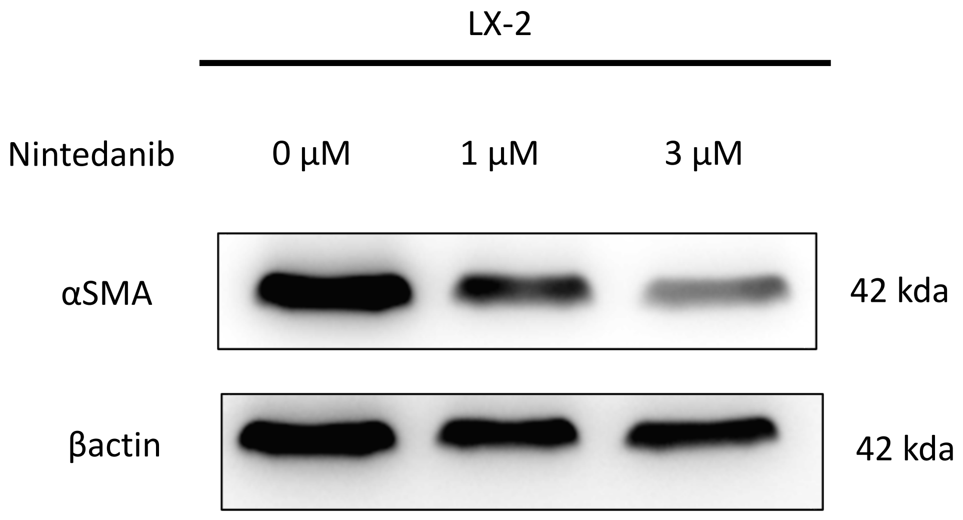

Supplementary table 1

105 types of Cytokines and Proteins			
**Interleukins**	**Chemokines**	**Growth factors**	**Others**
IL-1 alpha/IL-1F1	CCL2/MCP-1	FGF basic	IFN-gamma
IL-1 beta/IL-1F2	CCL7/MCP-3	KGF/FGF-6	M-CSF
IL-1ra/IL-1F3	CXCL9/MIG	FGF-18	G-CSF
IL-2	CCL3/CCL4 MIP-1 alpha/beta	EGF	GM-CSF
IL-3	CCL20/MIP-3 alpha	HGF	TNF-alpha
IL-4	CCL19/MIP-3 beta	PDGF-AA	Leptin
IL-5	CXCL10/IP-10	PDGF-AB/BB	BAFF/BLyS/TNFSF13B
IL-6	CXCL11/I-TAC	VEGF	Adiponectin/Acrp30
IL-8	CXCL4/PF4	TGF-alpha	Angiogenin
IL-10	CCL5/RANTES		Angiopoietin-1
IL-11	CXCL12/SDF-1 alpha		Angiopoietin-2
IL-12 p70	CCL17/TARC		Apolipoprotein A1
IL-13	CXCL5/ENA-78		BDNF
IL-15	CXCL1/GRO alpha		CD14
IL-16			CD30
IL-17A			CD31/PECAM-1
IL-18 BPa			CD40 Ligand/TNFSF5
IL-19			Chitinase 3-like
IL-22			Complement Component C5/C5a
IL-23			Complement Factor D
IL-24			C-Reactive Protein/CRP
IL-27			Cripto-1
IL-31			Cystatin C
IL-32 alpha/beta/gamma			Dkk-1
IL-33			DPPIV/CD26
IL-34			Endoglin/CD105
			EMMPRIN
			Fas Ligand
			Flt-3 Ligand
			GDF-15
			Growth Hormone (GH)
			ICAM-1/CD54
			IGFBP-2
			IGFBP-3
			Kallikrein 3/PSA
			LIF
			Lipocalin-2/NGAL
			MIF
			MMP-9
			Myeloperoxidase
			Osteopontin (OPN)
			Pentraxin 3/TSF-14
			RAGE
			RBP4
			Relaxin-2
			Resistin
			Serpin E1/PAI-1
			SHBG
			ST2/IL1 R4
			TFF3
			TfR
			Thrombospondin-1
			TIM-1
			uPAR
			VCAM-1
			Vitamin D BP

Supplementary Figure legends

Supplementary Figure 1. *In vivo* experimental schedule

(A) Comparison between HuCCT1 cells alone and HuCCT1 plus CAFs cells.

(B) Nintedanib and gemcitabine treatment schedule.

Supplementary Figure 2. Effects of CAF and nintedanib were observed in the supernatants of CM after ultracentrifugation

(A) The proliferation-enhancing effect and nintedanib treatment effect on HuCCT1 and RBE cell lines were observed only in the supernatants of CAF-CM and nintedanib-treated CAF-CM after ultracentrifugation.

Supplementary Figure 3. IL-6 and IL-8 promotes phosphorylation of STAT3 in ICC cells

(A) The effects of IL-6 and IL-8 on the phosphorylation of STAT3 in HuCCT1 were evaluated by Western blotting. IL-6 and IL-8 promoted the expression of the phospho-STAT3 in HuCCT1.

(B) With recombinant IL-6 and IL-8, the effects of nintedanib-treated CAF-CM on the proliferation and invasion of HuCCT1 were restored. (n = 5). *P < 0.01.

Supplementary Figure 4. Nintedanib treatment significantly reduced the number of phospho-STAT3 positive ICC cells

(A) The number of phospho-STAT3 in HuCCT1 was counted. Nintedanib treatment significantly reduced the number of pSTAT3 positive cells, with the greatest reduction observed with combined nintedanib and gemcitabine treatment. ($n = 5$). *$P < 0.05$.

Supplementary Figure 5. Body weight and serum biochemical tests of mice

(A) Relationship between mouse body weight and treatment group in the xenograft experiments. There was no correlation between the body weight and treatment group ($n = 3$).

(B) Effect of treatment on blood biochemical parameters. There was no apparent organ toxicity in each group. GEM, gemcitabine; ALT, alanine aminotransferase ($n = 3$).

Supplementary Figure 6. Effects of nintedanib on LX-2

Nintedanib also suppressed the proliferation and α-SMA expression of LX-2 ($n = 5$). *$P < 0.01$.

Supplementary table 1. 105 types of soluble factors, including cytokines and proteins
